# Paternal Postpartum Depression in South Asia

**DOI:** 10.1002/puh2.70281

**Published:** 2026-05-27

**Authors:** S. M. Yasir Arafat, Sadeed Hossain

**Affiliations:** ^1^ Biomedical Research Foundation Dhaka Bangladesh; ^2^ Department of Psychiatry Bangladesh Specialized Hospital Dhaka Bangladesh; ^3^ Department of Public and Community Health Faculty of Medicine and Health Sciences Frontier University Garowe Garowe Somalia

**Keywords:** Edinburgh Postnatal Depression Scale, father, paternal depression, postpartum depression

## Abstract

**Background:**

Postpartum depression (PPD) is a well‐recognized perinatal mental health condition, yet its occurrence among fathers remains poorly studied, particularly in South Asia where patriarchal norms obscure men's psychological distress.

**Objective:**

To examine the prevalence, assessment tools, risk and protective factors, and interventions for paternal PPD (PPPD) across South Asian countries.

**Methods:**

A search was conducted in PubMed, Scopus, Google Scholar, BanglaJOL, NepJOL, and Google using search terms. Research articles eligible for inclusion were original studies, quantitative analyses published in English from South Asian region. Descriptive analysis was performed considering the objectives.

**Results:**

This review included 13 articles from five South Asian countries published between 2015 and 2025. Sample sizes ranged from 15 to 826. The *Edinburgh Postnatal Depression Scale* was the most commonly used tool; the prevalence of PPPD ranged from 3% to 60%. Associated factors also varied from study to study.

**Conclusions:**

PPD among fathers has received academic attention in the recent decade in South Asia. Public health systems need to integrate paternal screening into maternal and child health programs, develop culturally validated tools, and train healthcare professionals to recognize gender‐specific presentations of depression.

## 1 Introduction

Postpartum depression (PPD) is characterized by depressive symptoms, including crying spells, insomnia, depressed mood, fatigue, anxiety, and poor concentration. It usually occurs within 4 weeks of delivery; however, it may develop as late as 30 weeks postpartum [1, 2]. Although PPD is widely studied in mothers, growing evidence indicates that fathers are also significantly affected. Approximately 25% of men in the community experience paternal PPD (PPPD) during the first postpartum year, with rates rising to 24%–50% when their partners are also affected [3, 4].

PPPD presents distinct clinical challenges. Unlike maternal depression, paternal depression often presents with atypical symptoms such as irritability, withdrawal, risk‐taking behavior, avoidance behavior, interpersonal conflicts, violence, and substance abuse, which makes diagnosis and screening very difficult [5, 6]. It can harmfully affect marital and partner relationships, impair father–infant bonding, and lead to poor child behavioral and emotional development [7]. Traditional gender norms, economic hardship, unemployment, low social support, and low marital satisfaction may lead to PPPD [8]. Studies have compared multiple screening instruments for paternal depression, including the *Edinburgh Postnatal Depression Scale* (EPDS), and *Patient Health Questionnaire*‐9 (PHQ‐9), and found that standard cutoff scores frequently require downward adjustment for men, and further adaptation for non‐Western cultural contexts [9, 10, 11]. Although PPPD negatively impacts family functioning and child development, it is an underscreened, underdiagnosed, and undertreated mental health issue [5, 7].

Approximately 2 billion people, making up nearly one‐fourth of the world's population, live in eight countries (Afghanistan, Bangladesh, Bhutan, India, Maldives, Nepal, Pakistan, and Sri Lanka) with low‐ and middle‐income country (LMIC) backgrounds in South Asia. This region is diverse, with many ethnicities, cultures, languages, religions, and political systems [12, 13, 14]. Gender preference, lack of access to mental health services, and cultural stigma around men's emotional vulnerability exacerbate the hidden burden of paternal mental health problems in South Asia [15, 16]. Considering the patriarchal social norms of this region, PPD among fathers has been poorly studied. Therefore, reviewing the available evidence on the topic would help to understand the burden, unmet need for services, and research momentum. It will also help to compare and contrast the metrics between the countries of the region. Therefore, this review aimed to examine the prevalence, assessment tools, risk and protective factors, and intervention strategies reported across studies of PPPD in South Asia. By mapping the current evidence, this review seeks to identify gaps, highlight methodological challenges, and generate actionable implications for research, clinical practice, and public health policy in the region.

## 2 Methods

### 2.1 Study Design

This study was conducted as a narrative review, aiming to synthesize existing evidence on PPPD in South Asia. Given a dearth of studies, significant heterogeneity in measurement tools, cutoff scores, and sample characteristics, a formal meta‐analysis was not feasible. The review is reported transparently and follows the broad principles of narrative synthesis.

### 2.2 Search Strategy

A comprehensive web‐based search was conducted without any time restrictions on publication date in PubMed, Scopus, Google Scholar, BanglaJOL, NepJOL, and Google using both Medical Subject Headings (MeSH) and free‐text terms to ensure sensitivity and relevance on September 06, 2025. Boolean operators were applied to formulate the search string:

(“paternal depression” OR “postpartum depression in fathers” OR “postnatal depression in fathers” OR “postpartum depression in men” OR “paternal postpartum” OR “postpartum paternal depression”)

AND

(“South Asia” OR “Afghanistan” OR “Bangladesh” OR “Bhutan” OR “India” OR “Maldives” OR “Nepal” “Pakistan” OR “Sri Lanka”).

### 2.3 Study Selection

The review draws on literature from the eight countries recognized as South Asian nations: Afghanistan, Bangladesh, Bhutan, India, Maldives, Nepal, Pakistan, and Sri Lanka. These countries share broadly comparable socioeconomic, cultural, and healthcare system characteristics, thereby making regional comparisons meaningful. Duplicate records were removed, and all retrieved studies were manually screened for relevance on the basis of titles, abstracts, and full texts.

#### 2.3.1 Inclusion Criteria

Articles eligible for inclusion were original studies and quantitative analyses available in full‐text form. Only peer‐reviewed articles published in English were included. The study was included if it focused on PPPD that occurred in eight South Asian countries.

#### 2.3.2 Exclusion Criteria

Studies were excluded if they were review articles, editorials, opinion pieces, qualitative studies, multiple reports published from the same data, or reports based solely on media coverage. In addition, studies on migrant populations residing outside the South Asian region were excluded to ensure geographic specificity.

### 2.4 Outcome Variables

From each included study, the following variables were extracted: authors, publication year, country, sample size, tools/instruments used, population characteristics, prevalence, associated factors, and key findings.

### 2.5 Data Analysis

Data were extracted independently and organized in a structured table using Microsoft Excel. Descriptive analysis was performed in accordance with the review objectives.

### 2.6 Ethical Considerations

This study used data from publicly available published academic literature. No human participants were directly involved in this study. Therefore, no formal ethical clearance was sought to conduct the review.

## 3 Results

### 3.1 Study Characteristics

This review included 13 articles from five South Asian countries published between 2015 and 2025 (Table 1). The majority were conducted in India (*n* = 5, 38.5%) [15, 17, 18, 19, 20] and Pakistan (*n* = 4, 30.8%) [21, 22, 23, 24], followed by Sri Lanka (*n* = 2, 15.4%) [25, 26], Bangladesh (*n* = 1, 7.7%) [27], and Nepal (*n* = 1, 7.7%) [28]. No study was identified from Afghanistan, Bhutan, and the Maldives. Sample sizes ranged widely from 15 [24] to 826 [28]. Most of the studies were cross‐sectional in design (*n* = 12, 92.3%), with one exception (cohort study) [19]. Among the included studies, six studies assessed PPD among both parents [15, 17, 18, 19, 25, 26], whereas seven studies assessed fathers only [20, 21, 22, 23, 24, 27, 28]. Eleven studies strictly reported PPD, except one Sri Lankan study [25], assessed perinatal rather than strictly PPD and one Indian study that assessed PPD following live birth and stillbirth [19].

**TABLE 1:** Summary of studies on paternal postpartum depression in South Asia.

Sr. no.	Study	Country	Design	Sample size (fathers)	Tool (cutoff)	Prevalence (%)	Associated factors
1	Yesmin et al. [27]	Bangladesh	Cross‐sectional	461	EPDS‐10	47.7 mild, 35.2 moderate, and 17.1 severe	Not reported
2	Thilagavathy [20]	India	Cross‐sectional	122	EPDS‐10 (>9)	59	New responsibilities and lifestyle changes
3	Salian and Shah [18]	India	Cross‐sectional	64	EPDS (>10)	30	Fatigue and stress
4	Babu and Jacob [17]	India	Cross‐sectional	235	EPDS‐10 (≥13)	0	Socioeconomic stress, prenatal depression and anxiety, and unintended pregnancy
5	Goyal et al. [15]	India	Cross‐sectional	479	EPDS (≥11)	12.9	Infant gender bias (higher in fathers of girls)
6	Sarkar et al. [19]	India	Cohort	150	EPDS‐10	Stillbirth—18.1, live birth—6.7	Not reported
7	Adhikari et al. [28]	Nepal	Cross‐sectional	826	PHQ‐9 (≥10)	3	Not reported
8	Noorullah et al. [23]	Pakistan	Cross‐sectional	120	EPDS‐10 (>10)	28.3 (mild 25.8; severe 2.5)	Lack of paternity leave
9	Yousef et al. [24]	Pakistan	Cross‐sectional	15	EPDS	60	Cultural role expectations, exclusion from caregiving, and work‐family imbalance
10	Atif et al. [22]	Pakistan	Cross‐sectional	51	EPDS‐10 (>10)	23.5	Maternal PPD and poor sleep
11	Abbasi et al. [21]	Pakistan	Cross‐sectional	400	EPDS‐10 (≥10)	Not reported	Neuroticism, younger age, younger spouse, and private employment
12	Siriwardhana et al. [26]	Sri Lanka	Cross‐sectional	435	EPDS (>7)	10.9	Maternal PPD, income decrement, and increased time at home
13	Hapangama et al. [25]	Sri Lanka	Cross‐sectional	181	DASS‐21	17 (perinatal)	Not reported

Abbreviations: DASS, Depression, Anxiety, and Stress Scale; EPDS, Edinburgh Postnatal Depression Scale; PHQ, Patient Health Questionnaire.

### 3.2 Assessment Tools and Cutoffs

The EPDS was the most commonly used tool, applied in 11 studies; however, cutoff scores were different (>7, >9, >10, ≥13). EPDS is a 10‐item self‐report scale that has a very simple method of scoring and can be completed within 5 min. Each item is rated on a 4‐point scale (from 0 to 3), and the total score ranges from 0 to 30. Commonly used cutoff points are 10–13 and with increasing scores indicating a greater likelihood of depressive symptoms [17, 29, 30]. Other instruments were also used, like the PHQ‐9 in Nepal [28] and the *Depression, Anxiety, and Stress Scale*‐21 (DASS‐21) in Sri Lanka [25].

### 3.3 Prevalence of PPPD

**FIGURE 1:**
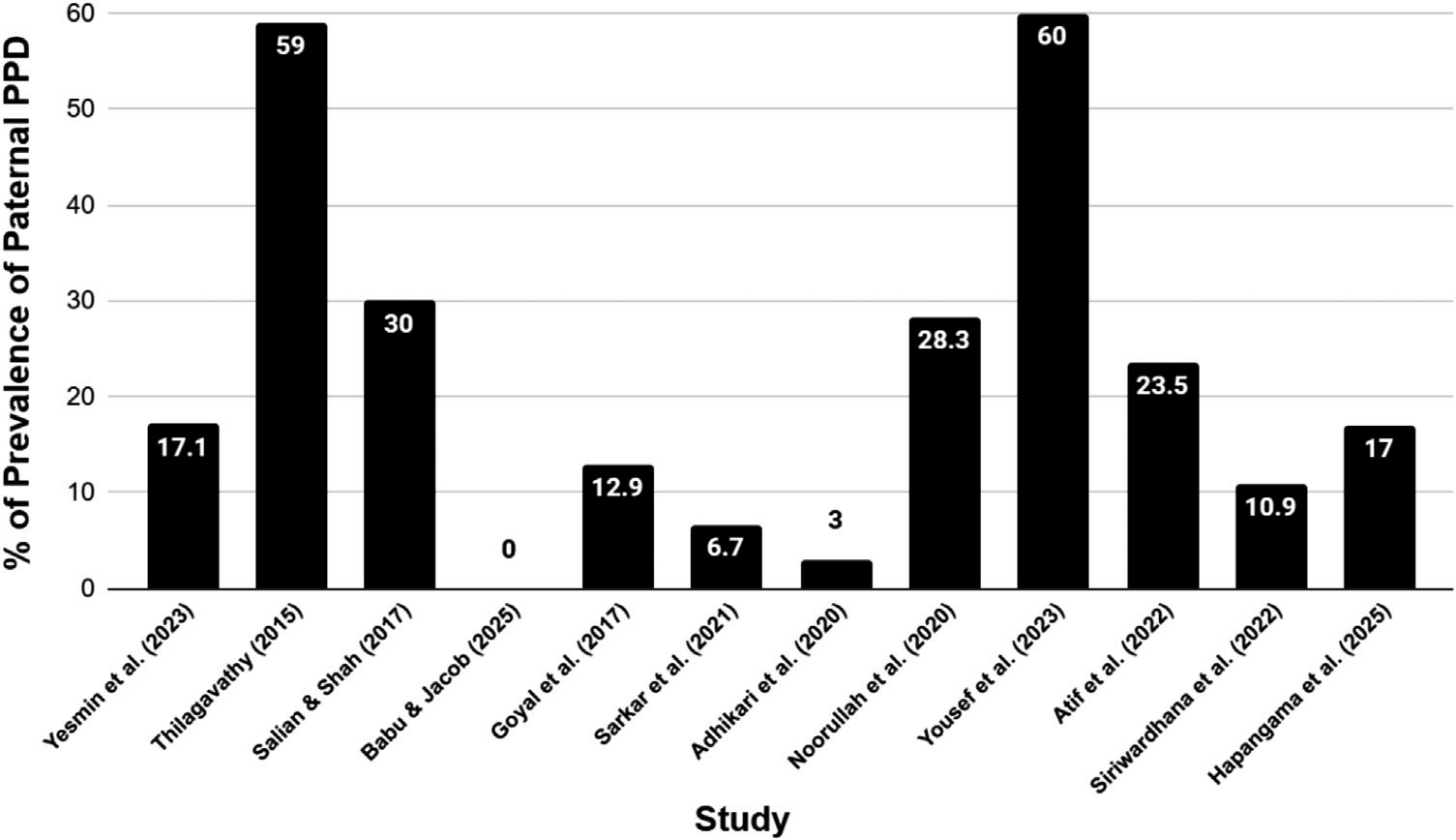
Prevalence of paternal postpartum depression in South Asia.

Prevalence of PPPD was variable in different studies (Figure 1). The highest prevalence (60%) was reported in a Pakistani study where sample size was 15 [24], whereas the lowest (3%) was observed in a Nepali cross‐sectional survey with a sample size of 826 [28]. Studies from India reported prevalence ranging from 0% to 59%, depending on sample type, cutoff scores, and timing of assessment. For example, Thilagavathy [20] found that 59% of first‐time fathers screened positive 4 to 5 months postpartum, whereas Babu and Jacob [17] used the EPDS cutoff of ≥13. In Bangladesh, Yesmin et al. [27] reported high levels of depressive symptoms, with 47.7% mild, 35.2% moderate, and 17.1% severe cases. Studies from Sri Lanka also found high prevalence of paternal PPD: 10.9% [26] and 17% [25]. When comparing screening tools, studies employing the EPDS generally reported higher prevalence rates, whereas those using the PHQ‐9 (Nepal, 3%) and the DASS‐21 (Sri Lanka, 17%) showed lower estimates.

### 3.4 Associated Factors

Younger paternal age, younger spouse age, limited time spent at home, and private employment status were associated with paternal depression in Pakistan [21]. Maternal PPD was also an important factor of paternal depression in both Pakistani and Sri Lankan studies [22, 26]. Additional risk factors included infant gender bias (depression was more common among fathers of daughters in India [15]), financial stress, unintended pregnancy, exclusion from caregiving roles, poor sleep quality, and increased responsibilities that were also reported [17, 18, 20, 22, 23, 24, 26]. Most of the studies did not properly report or suggest protective factors or interventions; however, some studies suggested family support, screening, counseling, psychoeducation, psychotherapy, and antidepressants as beneficial methods [17, 20, 23, 27].

### 3.5 Cross‐Country Scenario

Prevalence of PPPD had variation across South Asian countries. Studies from Pakistan consistently reported higher prevalence rates, ranging from 23.5% to 60%, particularly in small‐sample‐sized studies [24], with associated factors such as paternal depression, maternal PPD, younger paternal and maternal age, and work‐related stressors. In contrast, Indian studies reported highly heterogeneous findings, from 0% (a large sample study with an EPDS cutoff of more than 13 was used) [17] to as high as 59% among first‐time fathers [20]. Infant gender bias (negative toward female babies) was a unique associated factor that was reported in an Indian study [15]. Sri Lanka demonstrated prevalence rates of 10.9% and 17%, with maternal depression, financial stress, and increased time spent at home highlighted as important factors. In Bangladesh, a single study was conducted, and it found alarmingly high levels of depressive symptoms (47.7% mild, 35.2% moderate, and 17.1% severe) among fathers [27]. Meanwhile, Nepal reported one of the lowest prevalences (3%), possibly due to the use of an alternative instrument (PHQ‐9), and had a very high sample (826) [28].

Overall, Pakistan and Bangladesh appear to have higher reported prevalence compared to Nepal and Sri Lanka, whereas India shows the widest variability.

## 4 Discussion

This review synthesized evidence from 13 studies across five South Asian countries and revealed a heterogeneous burden of PPPD in the region. Prevalence estimates varied widely, ranging from as low as 3% in Nepal [28] to as high as 60% in a small‐sample Pakistani study [24]. This picture suggests that PPPD is not only present but also potentially underrecognized in South Asia, which is similar to global perspective [10]. Although the extreme variability limits direct comparison, several analytical observations can be drawn.

### 4.1 Methodological Heterogeneity and Its Consequences

The large variation in prevalence (from 3% to 60%) in this review cannot be understood without considering how different the studies were in their methods. Three key factors explain this variation.

First, the type of screening tool used makes a big difference. Studies that used the EPDS generally found higher rates of depression compared to those using tools like PHQ‐9 (which reported 3% in Nepal) or DASS‐21 (17% in Sri Lanka). This is partly because EPDS is more sensitive for detecting PPD [31, 32]. However, it was originally designed for women, so using it for men is not straightforward. Research shows that men often need a lower cutoff score for accurate detection [29]. For example, one study found that many Asian studies had to reduce the cutoff to better identify depression in men. In this review, different studies used different cutoff scores (ranging from >7 to ≥13), which makes direct comparison difficult and increases variation [33].

Second, sample size affects the results significantly. The high prevalence of 60% reported in Pakistan came from a very small sample of only 15 participants [24], which makes the result unreliable and likely exaggerated. On the other hand, the lowest estimate (3% from Nepal) came from the largest study with 826 participants [28], which is more likely to provide a stable and accurate estimate. In general, smaller studies tend to produce more extreme and less reliable results.

Third, the timing of assessment after childbirth also varies across studies. Some studies measured depression at a specific time (like 4 to 5 months after birth), whereas others used broader or unclear time periods. This matters because paternal depression does not follow the same timeline as maternal depression, so measuring at different times can lead to different prevalence rates. These measurement concerns are not unique to South Asia; globally, similar challenges have been raised, and EPDS has faced significant validation difficulties across cultures and populations, with studies from Asian settings frequently requiring lower cutoff scores than originally recommended.

### 4.2 Cultural and Contextual Interpretation of Findings

Beyond methodological differences, some variation may reflect real cultural and social differences between countries. In Pakistan, consistently high prevalence rates and qualitative studies suggest a clear sociocultural pattern: strict masculine norms that discourage emotional expression, lack of paternity leave, fathers being left out of perinatal care, and heavy financial pressure on men [16, 34]. Fatima et al. also identified stigma, marital conflict, and strong patriarchal responsibility as additional stressors in a tertiary care setting [32]. These factors do not just relate to PPPD; they actively shape it by influencing which emotions men feel able to express and which they suppress.

In India, the wide variation in findings (0%–59%) may be due to the country's internal diversity, including differences in socioeconomic status, urban versus rural settings, and family structures. One important finding is that fathers of female babies may have higher depression. This is similar to findings from China, where mothers of female infants had almost three times higher risk of depression than those with male infants [35]. This shows how cultural son preference and gender discrimination can directly affect mental health.

Bangladesh has only one study [27], which reported high levels of depression across all severity categories (mild, moderate, and severe). This may indicate a true high burden, or it could be due to overestimation by the measurement tool. Nepal reported a low prevalence using the PHQ‐9 in a large sample, but this may be due to lower sensitivity of the tool rather than a truly low rate of PPPD. Therefore, it should not be assumed that PPPD is rare in Nepal.

Globally, PPPD affects up to about 25% of fathers [11, 36, 37]; however, some studies from South Asia, especially from Bangladesh, India, and Pakistan, reported much higher rates. This may reflect a real higher burden due to multiple stress factors, but it could also be due to differences in study methods that inflate results. Better designed studies are needed to clearly understand this.

### 4.3 Gendered Expressions of Distress and the Limits of Current Screening

An important issue not well explored in South Asian research is how depression appears differently in men. Most studies used tools designed to detect typical depressive symptoms. However, men often show distress in different ways, such as irritability, emotional numbness, risk‐taking behavior, substance use, and aggression, which are not well captured by standard scales [8, 9]. This creates both a clinical and theoretical gap. Research on men's lived experiences of PPD shows deep suffering, loneliness, and feelings of failure that are often missed by current tools [38]. Therefore, there is a need to develop screening methods that specifically capture male patterns of distress, instead of only adjusting cutoffs of tools designed for women.

### 4.4 Institutional and Professional Barriers to Identification

The under‐detection of PPPD in South Asia is not only because men avoid seeking help. It is also related to how healthcare systems are structured. Fathers are often not included in perinatal care, and healthcare providers receive little training on paternal mental health. Studies from high‐income countries show that clinicians may notice signs of distress in fathers but struggle to identify and manage it due to limited time, consultation structure, and gender‐related beliefs [39, 40]. These challenges are likely even greater in South Asia, where healthcare systems mainly focus on mothers, staff shortages are common, and mental health stigma is high. Without clear changes such as including fathers in care and training professionals about PPPD, proper screening and treatment will remain difficult to achieve.

### 4.5 Cross‐Country Patterns in Context

Beyond methodological variation, cross‐country differences likely reflect genuine sociocultural differences. In Pakistan, the high rates seem linked to factors such as strict masculine roles, lack of paternity leave, limited involvement of fathers in perinatal care, and financial pressure as the main provider [16, 34]. In India, the wide variation (0%–59%) likely reflects its large diversity in income levels, living conditions, and family structures. One important finding was that having a daughter increased depression among fathers, showing the role of cultural gender preferences [15]. In Bangladesh, only one study is available, but it reported very high levels of depression, suggesting a serious but still poorly understood problem [27]. Nepal reported a low rate (3%), but this should be interpreted carefully, as the tool used (PHQ‐9) in a large sample may have underestimated the true burden [28]. In Sri Lanka, two studies (10.9% and 17%) showed that maternal depression and financial stress are important contributing factors, similar to patterns seen in other countries [22, 25].

### 4.6 Associated Factors in the South Asian Context

The review highlighted some associated factors that might influence PPPD in South Asia. Similar factors were seen in many studies around the world that influence PPPD [11]. Like Indian, Pakistani, and Sri Lankan fathers, employment instability and unintended pregnancies were important risk factors for Japanese fathers too [37]. Although Nishimura and Ohashi [37] did not find any association between paternal and maternal depression, we found studies from Pakistan and Sri Lanka reporting otherwise [22, 26]. Similar results were seen in other studies too [11, 41]. Other recurrent factors like younger paternal age and poor sleep quality are also reported in studies from other countries [42, 43]. Notably, in India, the birth of a female child increased PPD, which was similar in another Asian country. In China, mothers of female infants showed 2.9 times higher risk of PPD compared to mothers of male infants, potentially reflecting cultural preferences for male children and negative family reactions to female births [35].

However, a European country (France) reported that young French mothers who conceived male children experienced reduced quality of life regardless of depressive state [44].

### 4.7 Neglect of Protective Factors and Interventions

Most of the studies (*n* = 9) reported associated factors of PPPD; nonetheless, only four studies [17, 20, 23, 27] explored protective factors or interventions. As more than 92% of the studies were cross‐sectional studies, we could not find out the effects of interventions, and unfortunately the only cohort study did not report about the effects of intervention. The same issue can be seen in a global context. A recent systematic review about interventions for paternal perinatal depression only found 10 published interventions addressing the PPPD [45].

### 4.8 Research and Policy Implications

**For research**: Future studies could prioritize longitudinal and prospective designs to establish causal pathways between risk factors and PPPD. Standardized, culturally adapted, and male‐validated screening tools should be developed and tested across South Asian populations, with attention to language, literacy, and gender‐specific symptom presentation. Multicountry comparative studies using harmonized methods would help disentangle genuine cross‐national differences from methodological artifacts. Research on protective factors, particularly social and family support mechanisms, is critically needed.

**For clinical practice**: Routine paternal screening could be integrated into existing maternal and child health programs at low or no additional cost. Healthcare professionals require training in recognizing the atypical and gender‐specific presentations of PPPD. Consultation environments need to be made more father‐inclusive, shifting from a paradigm in which fathers are peripheral to one in which they are recognized as active stakeholders in perinatal health.

**For policy**: Governments in South Asia need to formally recognize paternal mental health as a component of family health policy. Introduction or expansion of paid paternity leave, inclusion of paternal mental health indicators in national health surveys, and investment in public awareness campaigns that reduce stigma around men's emotional vulnerability are all actionable steps. The treatment of paternal depression could be understood as an investment not only in men's well‐being but in maternal mental health, couple stability, and optimal child development.

### 4.9 Strengths and Limitations

To the best of our knowledge, this is the first review article on PPPD in South Asia. The study highlighted not only prevalence patterns but also cross‐country differences in associated factors, instruments, and cultural contexts.

Despite these strengths, several limitations should be acknowledged. First, the majority of included studies were cross‐sectional in design (92.3%), limiting the ability to establish causal relationships or assess the long‐term impact of risk factors and interventions. Second, there was considerable heterogeneity in measurement tools and cutoff scores (EPDS, PHQ‐9, and DASS‐21), which complicates direct comparison of prevalence estimates across countries. Third, the small number of studies in Bangladesh, Nepal, and Sri Lanka (one or two studies each) prevents generalization of findings for these countries. Fourth, sample sizes varied widely, with some studies being underpowered (e.g., sample size of only 15 in [24]), which could inflate prevalence estimation. Fifth, protective factors and intervention strategies were underexplored, which limits the ability to propose evidence‐based prevention and treatment approaches for PPPD for South Asia. Finally, this was a narrative review, not a systematic review. Therefore, no PRISMA framework or formal risk‐of‐bias assessment was used, which may affect reproducibility and introduce selection bias. Due to differences across studies in tools, methods, and populations, a meta‐analysis was not possible, so the findings should be interpreted with caution.

## 5 Conclusion

PPD among fathers has received academic attention in the recent decade in South Asia with variations in prevalence, measuring tools, study sample, and associated factors. The wide variation in results reflects methodological variation, particularly the use of different screening tools, cutoffs, and sample sizes. Public health systems in South Asia need to acknowledge paternal mental health as an essential component of family well‐being. Integration of paternal screening into maternal and child health programs, validation of appropriate tools, and development of culturally appropriate interventions could reduce the hidden burden of PPPD and improve outcomes for fathers, mothers, and children.

## Author Contributions

Conception: S. M. Yasir Arafat. Data curation: Sadeed Hossain. Writing – original draft: Sadeed Hossain. Writing – review and editing: S. M. Yasir Arafat. All authors have read and approved the final version of the manuscript.

## Funding

The authors have nothing to report.

## Conflicts of Interest

The authors declare no conflicts of interest.

## Data Availability

Data sharing is not applicable to this article as no datasets were generated or analyzed during the current study.
